# Characterization of the complete mitochondrial genome of *Ageratum conyzoides*

**DOI:** 10.1080/23802359.2019.1676176

**Published:** 2019-10-12

**Authors:** Ze-Ping Luo, Li-Wei Pan

**Affiliations:** College of Chemistry and Bio-engineering, Hechi University, Hechi, PR China

**Keywords:** *Ageratum conyzoides*, mitochondrial genome, Illumina sequencing, phylogenetic analysis

## Abstract

As a pharmaceutical plant with multi-bioactivity, *Ageratum conyzoides* appears to be a valuable agricultural resource. In this study, the complete mitochondrial (mt) genome of *A. conyzoides* was sequenced through Illumina sequencing method, and the mt genome was recovered after *de novo* assembly and annotation. The results showed that the 219,198 bp mt genome has a total of 52 genes, including 30 protein-coding genes, 3 rRNA genes and 19 tRNA genes. The overall GC content of this mitogenome is 45.4%. By phylogenetic analysis using maximum-likelihood (ML) method, *A. conyzoides* showed the closest relationship with *Diplostephium hartwegii* in the family of Asteroideae.

*Ageratum conyzoides* L., Asteraceae, is an annual herbaceous plant with a long history of traditional medicinal uses in several countries of the world and also has bioactivity with insecticidal and nematocidal acitivity (Ming 1999). In this study, we finished and analysed the mitochondrial (mt) genome of *A. conyzoides* based on the next-generation sequencing method.

Plant materials of *A. conyzoides* sequenced in this study were acquired from medical plants garden in Guiyang University of Traditional Chinese Medicine (26°57′N, 106°72′W). This specimen and its total genomic DNA and were stored in the Key Laboratory of Miao Medicine, Guiyang University of Traditional Chinese Medicine with accession NO. AGC-20170608-265710672. Sequencing was done on an Illumina HiSeq2500 platform. The clean reads were assembled by SPAdes version 3.11.1 (Bankevich et al. 2012) with default settings.

We used four mt genomes from Asteroideae plants as seeds to collect assembled mt fragments that facilitated by using Exonerate alignment (Slater and Birney 2005) through comparison between published protein-coding genes and the total assembled contigs. To fill the gap, Price (Ruby et al. 2013) and MITObim version 1.8 (Hahn et al. 2013) were applied and Bandage (Wick Ryan et al. 2015) was used to construct the circular assembly path guided by these positions of long scaffolds. The complete sequence was primarily annotated by Plann (Huang and Cronk 2015) and Exonerate combined with manual correction. All tRNAs were confirmed using the tRNAscan-SE search server (Lowe and Eddy 1997). Other protein-coding genes were verified by BLAST search on the NCBI website, and manual correction for start and stop codons was conducted. This complete mt genome sequence was submitted to GenBank under the accession numbers of MN075945.

The mt genome of *A. conyzoides* is a typical circular structure of 219,198 bp with with a GC content of 45.4%. Thi*s* mt genome contains a total of 52 genes, including 30 protein-coding genes, three rRNA genes (*rrn*5, *rrn*18, and *rrn*26), 19 complete native mt tRNA genes.

Another 15 published complete mt sequences in asterids were collected from the Genbank database. Whole genome-wide alignments using HomBlocks (Bi et al. 2018) under Gblocks trimming method (Dereeper et al. 2008), resulting in 25,724 characters in total, including almost all whole or partial PCGs and rRNA genes. ModelFinder (Kalyaanamoorthy et al. 2017) was used to identify the most appropriate substitution model. Phylogenetic analyses of the concatenated sequences were performed using the maximum-likelihood (ML) method implemented in IQ-TREE version 1.6.6 (Nguyen et al. 2014) according to the optimal substitution models under the rapid bootstrap algorithm (1000 replicates). To test support for the branch points of each gene trees, nonparametric branch support tests based on the Shimodaira–Hasegawa-like approximate likelihood ratio test (SH-like aLRT) procedure were also performed in the same run. As shown in Figure 1, the phylogenetic positions of these 16 mt genomes were successfully resolved with high bootstrap supports across almost all nodes. *A. conyzoides*, belonging to the Eupatorieae, exhibited the closest relationship with *Diplostephium hartwegii* in the family of Asteroideae.

**Figure 1.**
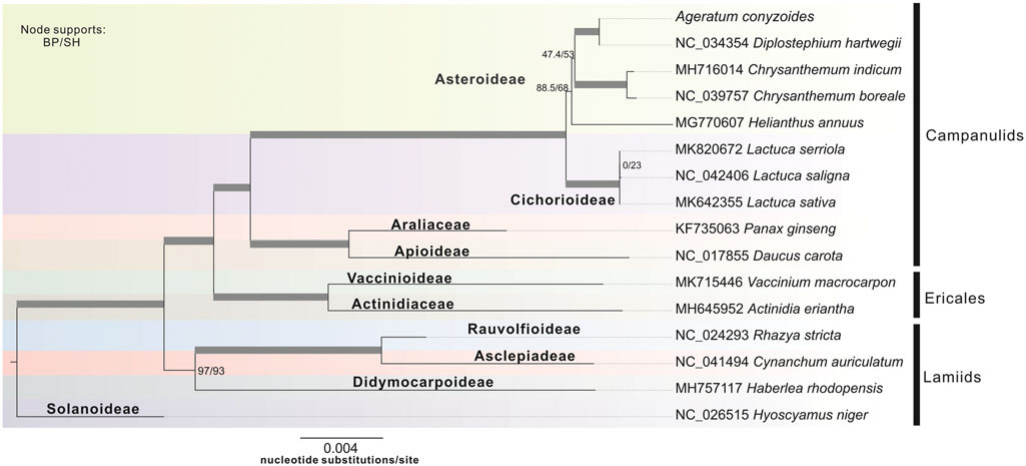
Phylogenetic tree yielded by ML analysis of 16 higher plant mt genomes. ML consensus tree is shown with bootstrap supports indicated by numbers besides branches. Branches harvesting both full bootstrap and SH-like aLRT values are indicated in bold. The scale indicates substitutions per site.
